# Identifying Barriers and Strategies to Support a Community Navigator-Driven Approach for Lung Cancer Screening

**DOI:** 10.3390/curroncol33090499

**Published:** 2026-08-24

**Authors:** Miranda J. Reid, Jennifer H. LeLaurin, Saba Ali, Caroline Sorial, Carma L. Bylund, Jennifer N. Woodard, Easton N. Wollney, Dianne L. Goede, Ji-Hyun Lee, Danielle S. Nelson, Lisa Carter-Bawa, Ramzi G. Salloum

**Affiliations:** 1 Health Outcomes and Biomedical Informatics, University of Florida, Gainesville, FL 32611, USA; 2 School of Medicine, University of Florida, Gainesville, FL 32611, USA; 3 University of Florida Health Cancer Institute, Gainesville, FL 32611, USA; 4 General Internal Medicine, University of Florida, Gainesville, FL 32611, USA; 5 Biostatistics, University of Florida, Gainesville, FL 32611, USA; 6 Community Health and Family Medicine, University of Florida, Gainesville, FL 32611, USA; 7 Center for Discovery and Innovation, Hackensack Meridian Health, Nutley, NJ 07110, USA

**Keywords:** lung cancer, lung cancer screening, implementation science, qualitative research, community navigator, patient navigation, shared decision making, decision aid

## Abstract

**Background/Objectives**: Although lung cancer is the leading cause of cancer-related deaths in the United States, rates of screening have remained persistently low nationwide. This study sought to identify barriers, facilitators, and support strategies necessary for implementing a novel community health navigator workflow to improve lung cancer screening uptake in both rural and urban settings. **Methods**: Semi-structured interviews were conducted with primary care providers (*n* = 5), community scientists (*n* = 7), community health navigators (*n* = 4), and radiology staff (*n* = 2). Interview transcripts were analyzed using a rapid qualitative analysis approach. Three authors coded based on the Consolidated Framework for Implementation Research (CFIR) and the Expert Recommendations for Implementing Change (ERIC) frameworks using a hybrid deductive–inductive approach. **Results**: Participants highlighted several primary barriers: access to knowledge and information (e.g., knowledge of eligibility, knowledge of insurance coverage), IT infrastructure (e.g., quality of pack-year data), relative priority (e.g., need to discuss other conditions), and patient needs and resources (e.g., time off work, transportation, difficulty scheduling). Key facilitators for screening were again IT infrastructure (e.g., automated electronic health record alerts) as well as relational connections (e.g., trust between patients and providers). To address provider-level barriers, participants recommended educational meetings, using clinical champions, and providing feedback on current lung cancer screening rates. To address patient-level barriers, participants recommended health education tools, providing transportation vouchers, hosting weekend lung cancer screening clinics, and assisting with scheduling. **Conclusions**: A community navigator approach to lung cancer screening should address key barriers to implementation on both the patient and provider level, including knowledge, prioritization, and patient access.

## Simple Summary

Lung cancer is the leading cause of cancer-related deaths in the United States, in part, due to consistently low screening rates. Interviews with health care providers and patients from rural and urban communities in North-Central Florida identified knowledge of lung cancer screening eligibility and insurance coverage, electronic health record infrastructure, and relative priority of screening as important barriers to lung cancer screening. Health care providers recommended holding educational meetings, using clinical champions and providing current screening rates to help improve provider knowledge and prioritization of screening. Patients recommended using health education tools, providing transportation vouchers, and assisting with scheduling to help improve patient knowledge and access to screening. These strategies may help improve lung cancer screening rates for patients in primary care clinics in both urban and rural areas.

## 1. Introduction

Lung cancer is the leading cause of cancer-related death in the United States, accounting for 127,070 deaths in 2023 [1]. Annual lung cancer screening with low-dose computed tomography (LDCT-LCS) is an important strategy for decreasing lung cancer mortality. To promote early identification of lung cancer, the US Preventive Services Task Force (USPSTF) released recommendations for annual lung cancer screening with low-dose computed tomography (LDCT-LCS) in 2013 and expanded them in 2021, by lowering the pack-year requirement and increasing the age range, overall increasing the eligible population by 50% [2,3]. Under the 2021 guidelines, adults aged 50 to 80 years with at least a 20 pack-year smoking history who are either current smokers or have quit within the past 15 years are eligible for LDCT-LCS [2]. Despite these guidelines, screening rates have remained low. In 2022, only 15.8% of eligible individuals received lung cancer screening in Florida [4]. If lung cancer screening rates rose from 15% to 80%, an estimated 110,000 lives and 1.5 million life-years could be saved in the U.S. between 2021 and 2045 [5].

One of the major facilitators of patient utilization of LDCT-LCS is a provider recommendation to screen [6,7], and most eligible individuals who receive a recommendation are willing to be screened [8,9]. However, most eligible individuals have never received a recommendation for LDCT-LCS from their provider [10,11]. This is in part because, unlike other routine health screenings that are age-based, lung cancer screening is only relevant to a narrower group of patients with a specific smoking history. Properly collecting smoking history is a nuanced task, challenged by rigid electronic medical record (EMR) input requirements. Given inconsistent documentation of smoking history in the EMR [9,12,13,14], providers are often unaware that an individual patient is eligible. Patients themselves are often unaware that a screening test for lung cancer exists [15,16], which may prevent them from introducing it with their provider. In addition to a lack of provider and patient awareness of eligibility, time pressure during the short length of covered visits and the need to discuss other health concerns in that same visit are also important barriers to discussion of LDCT-LCS [6,10,12,17,18,19,20]. To address these barriers, the University of Florida (UF) Health Cancer Institute is piloting a clinical community navigator-driven referral system to improve uptake of LDCT-LCS in five primary care clinics. As part of this pilot, the clinical community navigators will reach out to patients through the EMR, share health education materials, including LungTalk, a computer-tailored decision support tool designed to facilitate shared decision making about lung cancer screening [21,22], and help patients navigate barriers to scheduling and attending LDCT-LCS.

The purpose of this study was to prepare for this pilot by identifying patient-, provider-, and system-level barriers and facilitators to the uptake of LDCT-LCS in urban and rural settings. Through qualitative interviews, we assessed barriers and facilitators and solicited feedback on the proposed pilot, as well as complementary implementation strategies to support the adoption of LDCT-LCS. Findings from this qualitative analysis informed adaptations to the clinical community navigator pilot and development of an implementation support plan.

## 2. Materials and Methods

We conducted semi-structured interviews with providers, staff, and community scientists between June and August 2025. We analyzed interviews with hybrid deductive–inductive coding using a rapid qualitative analysis approach to evaluate barriers, facilitators, and strategies to support uptake of lung cancer screening at primary care sites [23,24]. The study was determined to be exempt by the UF institutional review board (ET00047030). Reporting is guided by the framework for Planning for and Assessing Rigor in Rapid Qualitative Analysis (PARRQA) [25].

### 2.1. Participant Recruitment

We selected interview participants to represent provider, staff, and patient perspectives from both urban and rural settings. Providers and staff were recruited from UF Health to represent each step of the referral pathway (i.e., primary care providers, clinical community navigators, and radiology staff). Clinician partners emailed invitations to participate in the study to relevant providers and staff. To provide a patient and community member perspective, community scientists were recruited from the UF Community Scientist Program. Members of the program are community members from the UF Health Cancer Institute catchment area. As part of the program, they complete training in research methods and ethics and regularly consult on research studies to provide a lay perspective [26]. The program administrator sent introductory emails inviting community scientists to participate in the study. Interviews were completed on a rolling basis, concurrent with data analysis, until thematic saturation was reached. After the initial round of interviews, participants were recruited purposively to capture perspectives that had higher variability in experience in initial interviews (i.e., rural perspectives and community scientist perspectives). Recruitment ended once no additional sub-themes emerged during interviews.

### 2.2. Data Collection

**Figure 1:**
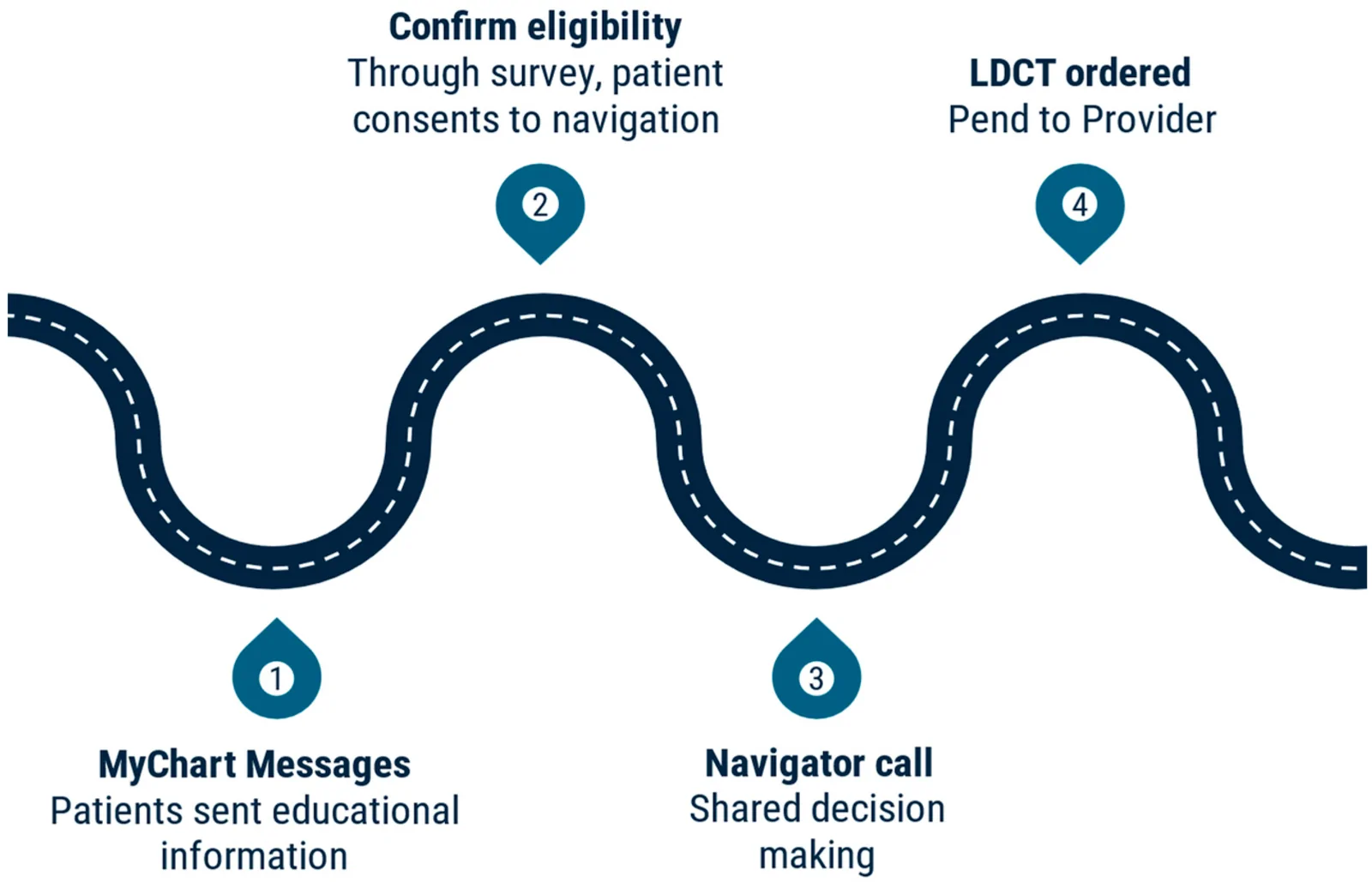
Proposed community navigator workflow presented to interview participants.

Two interview guides were developed, with one designed for community scientists and one designed for providers and staff (see Supplementary Materials). Both guides were designed to identify barriers and facilitators to lung cancer screening uptake, obtain feedback on the proposed intervention (Figure 1), and assess the appropriateness of implementation strategies. In the proposed intervention, the clinical community navigators will reach out to patients through the EMR, share health education materials, including LungTalk, have a shared decision-making conversation, and then help providers place LDCT orders for patients who are both eligible and interested. LungTalk is a multi-media, computer-tailored decision support tool grounded in the Conceptual Model on Lung Cancer Screening Participation. It is designed to facilitate shared decision making about lung cancer screening by increasing knowledge about screening and its risks and benefits, in a manner tailored to a patient’s smoking status [21]. Guides were open-ended with prompts informed by the Consolidated Framework for Implementation Research (CFIR) and the Expert Recommendations for Implementing Change (ERIC) [27,28,29,30]. CFIR is a determinant framework used to identify barriers and facilitators that influence implementation success. ERIC is a compilation of implementation strategies made to help standardize language in this field for easier replication. Implementation strategies with stronger evidence in the literature, such as “audit and provide feedback” and “prepare patients to be active participants”, were prioritized for inclusion in the interview guide [31]. Community scientists were asked to review strategies to address patient-facing barriers, while providers and staff were asked to review strategies to address provider- and health system-facing barriers. Interviews lasted 20–45 min and were audio-recorded using web-based meeting software (Zoom) and transcribed verbatim. Interview participants received a $25 electronic gift card. Participants verbally consented prior to initiation of audio-recording.

### 2.3. Data Extraction

Interview transcripts were analyzed with hybrid deductive–inductive coding using a rapid qualitative analysis approach [23,24,32]. A rapid qualitative analysis approach is often used in health services research, as it produces similarly rigorous results to more traditional qualitative analysis in a timeline that allows for the results to be incorporated into intervention design [33,34,35,36]. As part of this approach, data from each interview was summarized into a template based on the interview guide [32]. To pilot test the template, all three coders summarized the first two interviews and then met to come to consensus. To ensure dependability, subsequent interviews were summarized independently by two coders, and any conflicts were resolved by group discussion until consensus was reached [37].

### 2.4. Matrix Analysis

Data were transferred into a respondent by domain matrix in Microsoft Excel [38]. As the interview guides were based on existing frameworks, the matrices were then coded with a hybrid deductive–inductive approach using thematic content analysis aligned with the CFIR and ERIC frameworks [24,27,28,29,30,39,40]. All three coders coded the respondent by domain matrix with responses from the first eleven interviews; coding was not limited to preliminary codes with inductive codes assigned to data that described further concepts [24]. Coders then met to come to consensus and revise the codebook, with inductive codes from individual coding mapped onto additional CFIR or ERIC constructs where appropriate. For the remaining interviews, two coders coded each response in the matrix, with coders memoing and meeting regularly to reach consensus during data synthesis [25]. After each meeting, additional codes were added and previously coded responses were back-coded as necessary. After coding was completed, three researchers met and reviewed the coded matrices to develop themes.

## 3. Results

Eighteen participants (5 primary care providers, 4 clinical community navigators, 2 radiology staff, and 7 community scientists) were interviewed. Participants were majority female (*n* = 12), and age ranged from 23 to 74 years. Primary care providers ranged from 7 to 28 years in practice. Participants identified barriers at the provider, patient, and organizational levels and recommended corresponding implementation strategies (Table 1). Community scientists and health care providers (i.e., primary care providers, clinical community navigators, and radiology staff) were largely aligned on barriers, facilitators, and strategies. All barriers and facilitators described below were mentioned by both groups. As community scientist interviews were more focused on strategies to address patient-facing barriers, they were not specifically prompted with all possible provider-facing strategies. Two provider-facing strategies (i.e., identifying and preparing clinical champions, auditing and providing feedback) were identified in health care provider interviews, but not in community scientist interviews. All remaining strategies described below were identified by both groups.

### 3.1. Logistical Barriers to Screening

Participants identified a number of logistical barriers that prevent patients from completing their lung cancer screening. These barriers spanned constructs in the CFIR outer setting (i.e., patient needs and resources) and inner setting domains (physical infrastructure, IT infrastructure, work infrastructure). Participants suggested several strategies to address these barriers, which largely fit under the ERIC strategies of addressing patient access and assessing and redefining workflows.

#### 3.1.1. Physical Infrastructure

**Figure 2:**
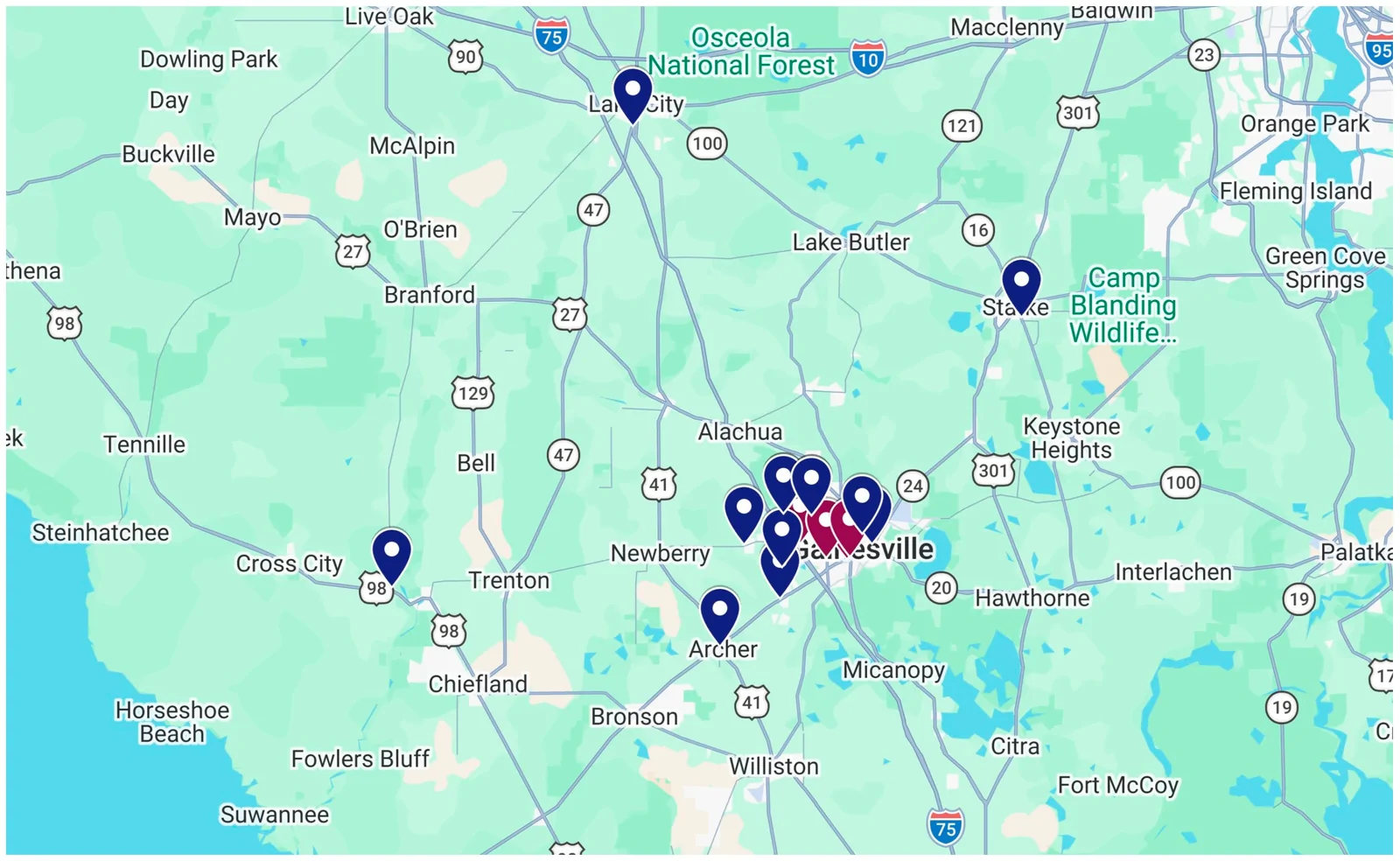
Locations of UF Health CT scanners available for patient screening (magenta) and UF Health primary care clinics (blue) in the UF Health Cancer Institute catchment area.

Participants acknowledged the limited locations available to UF Health patients for LDCT as a concern. All North-Central Florida locations are within the city of Gainesville, and two of the four CT scanners are on the main hospital campus, which is an 1100-bed hospital across six buildings (Figure 2). This was perceived to limit the access of rural residents in particular, with one community scientist stating:

“*The furthest town in Levy County is over an hour away from Gainesville … [It’s] difficult you know for some to get from that part of Levy County to Gainesville for the different tests and … people just kind of ignore their signs, because they think it’s gonna get better. It’s gonna clear because they can’t get to where they need.*”

Participant H, Community Scientist

One participant elaborated that it was not simply the distance that decreased patient willingness, but that almost all of the CT scanners were located in an urban environment. This provider suggested that the one suburban scanner might be more appealing, as it was both closer to the rural clinics and easier to navigate:

“*With your average asymptomatic [rural clinic] patient who’s maybe on disability doesn’t have a very good car, would have to pay their neighbor 20 bucks one way to take them into Gainesville? Yeah, it’s a high bar to convince that person to do something… I mean people in [rural town] don’t like going to Gainesville almost without exception. They hate it. They hate going to Gainesville. I can sometimes I say, ‘OK, well, you know where the [suburban mall] is- you’re not going to hit the bad part of traffic. It’s easy to park, easy to get in and out.’ I can use that to my advantage. If I’m telling them to go all the way to the hospital, to deal with the parking nightmare, the confusion of our buildings and where radiology is and this that, and the other. I mean, it’s a very, very high bar to convince somebody to want to do that*.”

Participant M, Primary Care Provider

#### 3.1.2. Patient Needs and Resources

In addition to the patient barriers under physical infrastructure, participants suggested that transportation and time off work could be key patient barriers. Transportation overlapped with concerns over cost and distance, with one community scientist describing how limited transportation options in both rural and urban areas are compounded by cost concerns: “*If you do find one of the transport companies, if they don’t take, you know, Medicaid, …then they may not have the funds to even pay for the transport companies to take them to places where they’re needed.*” A community scientist also described how their previous health education events had had limited reach, as they were only hosted during work hours: “*They asked that it could be on the weekends for those individuals who work. For the younger individuals and such like that, because the most people that were turned out during the week are people that are retired.*” Participants were similarly concerned that only offering screening during work hours would limit people’s ability to participate.

#### 3.1.3. Addressing Patient Access

To address distance and transportation barriers, participants suggested mobile screening units, such as those available for mammography, and travel vouchers to reimburse transportation or gas costs. Extending hours for CT screening was another popular suggestion, as participants had seen that be successful in other initiatives. One clinical community navigator compared expansion of MRI hours, stating, “*Whenever MRI opened up to doing screenings later in the evening and on the weekends, patients tended to appreciate that a lot. I think it’ll be the same way with CT scans that they’ll get it quite a bit.”* Additionally, clarifying insurance coverage for providers and patients was seen as important for ensuring access. One community scientist stated that all materials sent to patients should make that clear from the beginning:

“*I think it needs to be made clear from step one that this is free… I think a lot of people think CT, they think money, copay, they’re not seeing the screening part of it. Traditionally screenings are free, [but] because … they don’t understand that and … we can help with that.*”

Participant O, Community Scientist

#### 3.1.4. IT Infrastructure

The EMR was seen as both a barrier and facilitator for patients and providers, with some providers pointing out that the current reminders were a key part of their workflow for patients who they were able to refer for screening. One primary care provider discussed how this was especially helpful given the recentness of the lung care screening recommendations compared to other screenings, stating:

“*The [EMR health maintenance tab] including lung cancer screening is a huge value. I think that otherwise it very well could get lost in the shuffle [with] lung cancer screening being semi-new to the game in terms of cancer screenings. [It] continues to be the one that you have to really kind of remember.*”

Participant C, Primary Care Provider

Other providers ran into issues with documenting smoking history in the EMR and ordering lung cancer screening tests. One provider specifically mentioned that there was a “moment in time where [the LDCT-LCS order] was still on the old guidelines” and providers would have to confirm that patients fit within the narrower 2013 USPSTF criteria in order to send the order. Providers repeatedly mentioned how the reminder to screen did not show up for all eligible patients, describing how if smoking history was not documented in the exact right place and with the exact correct formatting, the screening would not show up in the health maintenance tab, with one provider saying:

“*If you don’t input the pack years exactly correctly in the EMR, it doesn’t show up correctly … and also the way that you put in the smoking history is really weird in the EMR because you have to put in date by date, how many cigarettes? … It’s supposed to auto calculate pack years, but it doesn’t do it perfectly*.”

Participant J, Primary Care Provider

Participants were also mixed on whether messaging patients through the EMR would be able to reach all the potentially eligible patients, with special concern for older adults, with one primary care provider stating, “*So you’re missing a lot of patients that are older that may not use [the EMR] and rely on like their phone, like phone calls and things like that*.” On the other hand, community scientists who were older themselves had fewer concerns about older adults using the EMR. A community scientist described his own use of the EMR and telemedicine to explain why he thought it would be feasible:

“*I have no issue with [EMR] cause I’m someone who used that, even though I’m kind of old. I mean, I’m not old, old, but I mean, so I’ve no qualms with telemedicine that kind of use of portals and EMR data. … I use those all the time with my primary care physician, … [it’s] almost 90% of our communications*.”

Participant K, Community Scientist

#### 3.1.5. Work Infrastructure

Insufficient staffing was seen as a major barrier to lung cancer screening. Both community scientists and providers mentioned the difficulty patients faced when trying to schedule their LDCT-LCS. One community scientist described the process of trying to schedule, saying, “*I spent more than an hour on the phone making calls to radiology to try and schedule one. The callback came but it was 3 h late and I’d left the house.” Similarly, a primary care provider stated that after putting in the order for screening “then somebody’s got to call and schedule… There’s a big fall off in that step*.” The radiology department described scheduling as “their biggest hurdle”, with one radiology administrator explaining that “We are very busy department so trying to get them in at on a reasonable amount of time can sometimes be challenging.” Radiology staff also explained that staffing limited their ability to expand hours, with one administrator saying, “*Currently, our outpatient schedules only run Monday through Friday. We don’t have Saturday, Sunday, outpatient schedules. We don’t have the staffing to support that right now.*” While extended hours may increase the uptake of LDCT-LCS, the department’s capacity limited the ability to address that need.

Staffing also impacted documentation of smoking status in the EMR. In some clinics, medical assistants were able to check smoking history while rooming the patient, but in many, this was highly variable, as they also needed to perform medication reconciliation and quickly room patients. One primary care provider described how staffing impacted that process:

“*I think the [medical assistants] have a lot to do when they’re rooming the patient, especially if the patient’s late and this and that. So it’s like a little bit of a, ‘Hey, do we spend a lot of time with the patient prior to the physician coming in or not?’ So I think staffing levels in terms of that, but like someone has to do the smoking history and who is it and how much time do you spend on it before the physician comes.*”

Participant J, Primary Care Provider

The variation from clinic to clinic meant that it was not always clear whose responsibility (medical assistant or primary care provider) it was to confirm smoking history at each visit.

#### 3.1.6. Assessing and Redesigning Workflows

Participants thought adding a clinical community navigator to both help identify patients and help them schedule and attend appointments would work well to address the work infrastructure and IT infrastructure barriers. Clinical community navigators had had success with similar workflows in other cancer screenings, with one clinical community navigator describing how helping with paperwork and scheduling had helped support patients in need of mammograms:

“*I think that like a large part of our job is taking over the logistics of getting people set up like even for example like mammograms, … We’re the ones scheduling it and updating them throughout … I think it just kind of keeps them informed throughout the process but also takes a lot of the like tasks that they need to do off of their back*.”

Participant E, Clinical Community Navigator

Community health workers in rural areas also added that they had been able to help patients navigate additional barriers, like internet access or technological literacy in order to get them to attend other screenings, with one clinical community navigator stating:

“*It’s a lot to get people where they need to be, and then once I get them going, they tend to do a lot better. You know, once I sit them down and say here this is how easy it is to use [EMR]. Oh, I never knew that it’s like well. Yeah, you can come up here anytime you need to. If you need to connect to the Internet, just come on up here and you know, so that does work pretty well. But it just takes a lot of reinforcement and a lot of knowing what the patient needs to do next.*”

Participant P, Clinical Community Navigator

Providers also thought having someone identify and reach out to patients about their eligibility, separate from a primary care visit, was a helpful additional workflow that would allow more patients to be screened. One provider described it as the “future of medicine”, saying:

“*I really think that this is the future of medicine that we are not doing enough of, whether it’s lung cancer, whether it is reminding people about mammograms, waiting for someone to come for their annual exam. And for me to remember to go through every one of the things is good, but it’s not perfect. I think having both your primary care doctor having it on their radar on part of their workflow is great, but separately, having the chart reviewed electronically identifying people, offering them a workflow I think is really important*.”

Participant F, Primary Care Provider

### 3.2. Difficulty Prioritizing Lung Cancer Screening

Participants identified the CFIR inner setting construct of relative priority as a barrier for both patients and providers. Participants suggested that maximizing the CFIR inner setting construct of relational connections, which was seen as a major facilitator, could help patients prioritize LDCT-LCS. For providers, the ERIC strategies of identifying and preparing clinical champions and of auditing and providing feedback were perceived as effective ways to raise the prioritization of LDCT-LCS.

#### 3.2.1. Relative Priority

Participants discussed how primary care visits have a range of goals, including treatment of new acute issues, chronic condition management, and primary and secondary prevention efforts. Providers discussed how LDCT-LCS can fall to the bottom of that list given that major societies were reluctant to recommend it for many years. One provider suggested that the USPSTF’s decision to rate it as a B recommendation, as opposed to the higher-grade A recommendation given to recommendations with the strongest evidence (e.g., cervical, breast, and colorectal cancer screening), impacted provider’s prioritization, stating:

“*It’s a level B recommendation, so … there’s probably some clinicians that it’s not on the top of their priority list. There’s a lot of stuff to do in primary care, and so if it’s not for some reason, really flashing red, you know, it’s easy to lose track of stuff. I mean, I think that’s just a general thing you’ll hear from any primary care doctor ever.*”

Participant M, Primary Care Provider

Providers also described how they often perceived patients to disproportionately prioritize acute and chronic condition management over screening, which makes it difficult for them to fit in all the screenings within a brief visit, with one provider stating:

“*I come to the table wanting to do all of the screening things that are appropriate for our patient and go through all of the things that can promote health. Patients walk into their annual exam and their expectations are very different. It is very often that they walk in and say, ‘OK, I’m here for my annual. I’ve been having headaches. My ears seem to ring. Sometimes my elbow bothers me occasionally. [I] twisted my ankle 4 weeks ago, it seems like it’s feeling better, but every once in a while it bothers me. I’ve got dry eyes.’ And their idea of an annual exam is the 40 or 50 things they experienced over the past year that they want to address. Whereas I’m looking at health promotion disease prevention, and so it is very hard to balance their expectations and the time it takes.*”

Participant F, Primary Care Provider

#### 3.2.2. Relational Connections

For patients, participants thought emphasizing the strong relationships that patients already have with the health system and with their providers could help patients prioritize screening. One community scientist emphasized the good will the health system has built up in rural areas saying, “*We know [health system] is here, we know it’s a phenomenal institution.*” Other participants thought the relationship between patients and their providers was even stronger and so any messaging to patients should be tailored to message their specific primary care provider. One clinical community navigator described the difference between how patients react to messages from the health system more generally versus from their provider:

“*The other thing is patients here really like their doctors. So if I say hi, I’m calling from [health system] … they may not even listen to me, but if I say hey, I’m calling from Doctor [Name], he wanted me to follow up with you on this X-ray. Ohh. OK. Well, you know, it’s a total difference on how they receive that information.*”

Participant P, Clinical Community Navigator

#### 3.2.3. Identifying and Preparing Clinical Champions

For providers, participants thought that having another provider on board as a champion, even if they were from a different clinic, would help them prioritize that initiative among the many quality improvement and research projects happening at the institution. One provider mentioned how having a clinical champion for a hormone replacement therapy initiative had served as an easy point of contact for questions, allowing them to prioritize that initiative:

“*We are a huge department, but we are pretty close, so if we know like this person loves hormone replacement therapy and even if there’s not a champion in my office, I’m like I’m going to go to a person in [other clinic] and just shoot them a quick message. So even if there wasn’t an office person, a departmental person would be the next best thing*.”

Participant F, Primary Care Provider

#### 3.2.4. Auditing and Providing Feedback

Providers also reiterated the importance of collecting and sharing current screening rates. Without knowing the current screening rates, it was hard to prioritize LDCT-LCS, especially since they regularly received data on how they were doing across a range of other quality improvement metrics. One primary care provider stated, “*I feel that we screen pretty well and I don’t know what our goal is and how far away we are from that goal.*” While institutional data suggested that their clinic had a low screening rate, none of that data had been shared with providers, so many felt they were screening most eligible patients. One provider elaborated on how comparative data in particular could help them prioritize LDCT-LCS:

“*Iterative feedback is really important, whether it’s on a personal level or clinic level. I think it would be very helpful for our clinic to see, you know, for the six months, January to June the year prior. You guys had done, you know, 26 referrals for lung cancer screening with the new initiative you guys have done 80. …It’s not just you referred 6 people last month. It’s you did 6 … but every one of your peers did 34 like those are the kind of things that make you say, man, am I taking as good a care of people as I should?*”

Participant F, Primary Care Provider

### 3.3. Knowledge of Lung Cancer Screening

Another important inner setting barrier was access to knowledge and information, which overlapped with the outer setting barrier of financing. Participants believed that eligible patients were largely unaware that lung cancer screening exists. Participants suggested that providers were generally aware of lung cancer screening but could potentially use reinforcement on the specifics of current guidelines and insurance coverage. Participants recommended strategies that generally aligned with the ERIC strategies of conducting educational meetings and raising patient awareness of the intervention.

#### 3.3.1. Access to Knowledge and Information

Participants suggested that most eligible patients may not know lung cancer screening existed, with one clinical community navigator conceding that the first barrier to patients may be knowledge: “*The first thing that comes to mind is like knowing that there even is a lung cancer screening*.” Other participants described fatalistic beliefs about lung cancer prognosis that may discourage patients from pursuing screening. One community scientist stated, “*I think people still see lung cancer as if you have it, you will die tomorrow… ‘Oh lung cancer. OK, you know when’s the funeral scheduled?*’” Another community scientist clarified that there is a lack of awareness of LDCT-LCS compared to other screenings, saying:

“*I think part of it is going to be education because people know- mammograms are drilled into our brain now, so we got mammograms down to some extent. Men’s PSA testing is kind of there, but a lot of people probably don’t realize that they can use radiology and determine if they have a lung cancer screening situation.*”

Participant K, Community Scientist

#### 3.3.2. Financing

As patients within the health system’s primary care clinics are typically insured, participants did not think cost would directly be a challenge once tests were ordered. However, they did point out that for those outside the health system this was an important barrier, with one community scientist stating, “The big asterisk is as long as you’re insured … When you’re insured, they’ll always they’ll keep a spot for you.” Additionally, participants suggested that confusion around insurance coverage between insurers could make primary care providers less likely to recommend screening in certain cases, with one primary care provider stating, “Medicare doesn’t pay for the same patients that the USPSTF recommends.” Several providers pointed out this difference in coverage, and they themselves were often unsure of which ages Medicare did not cover, with some mentioning ages 50–55 and others stating ages 75–80 (Medicare covers screening for ages 50–77. All private insurers are required to cover the entire USPSTF recommended age range of 50–80). Similarly, participants suggested that patients may not be aware that their insurance covers lung cancer screening without patient copays. One clinical community navigator compared it to her experience with helping patients get screened for breast cancer:

“*I mean, some people might not realize that their insurance covers it or how much their insurance does, even with, like, mammograms. Some patients have been like, ‘I want to at least, like, postpone this mammogram, because I don’t know if I have to pay for it.’ Even though, like, insurance companies are required to pay for it, like cover the screening mammogram once every year, once every two years. It’s just an understanding of like, how insurance works.*”

Participant G, Clinical Community Navigator

#### 3.3.3. Conducting Educational Meetings

To address awareness on the provider side, participants suggested incorporating educational presentations into routine clinic meetings. One provider thought this strategy could help raise awareness of both LDCT-LCS more generally and any specific interventions, saying:

“*I think coming to the monthly clinic meetings is pretty important. It builds trust as a team member…It serves the dual purpose of providing reinforcing education on lung cancer screening guidelines … and discussing the implementation of the project.*”

Participant C, Primary Care Provider

#### 3.3.4. Raising Patient Awareness of Intervention

To address patient knowledge of lung cancer screening, participants suggested a range of strategies to raise awareness. Some participants thought broader promotion through social media or community partners could be helpful. One community scientist suggested that in addition to local public health departments, partnering with churches and community organizations would be useful in rural areas:

“*Even though [health system] would come to these communities once a month. Not everyone knew, so they’re not- you’re coming, but you’re going to a health department that is no longer a primary care type health department…. So you’re tapping into a resource that’s not as visible themselves per se. So … connecting to the community organizers, the community leaders, the churches, the pastors who have that would be the key.*”

Participant H, Community Scientist

Generally, though, participants thought that since the eligibility criteria for LDCT-LCS are narrower than other cancer screenings, it would be more efficient to start by reaching out to patients who have been identified as eligible through the EMR. One community scientist summarized this view, saying, “Because you want to get such a specific age range, I think the energies and money put into the flyers, emails, and social media. It’s like hit or miss because it’s so broad.”

Participants were supportive of sharing decision support tools through the EMR, and in particular liked that the proposed tool, LungTalk, could be completed at home and provided an interactive, tailored experience. A clinical community navigator suggested that “*patients [will] feel like they’ve like they’re more cared for … when they have that more of that interaction and that will just make them … want to continue completing the screenings.*” In addition to sharing LungTalk, participants also thought some way of sharing other patients’ experience with LDCT-LCS could be useful. One community scientist who had helped develop attestations for other studies suggested:

“*There’s sort of a misconception about CT scans; and many people who have not had them … don’t realize how easy these scans are and how quick they are … I’ve been involved in some studies where patients have heard from other patients. But I’m wondering if either a couple of interviews with patients who’ve had the lung screening could be provided either by video or written template.*”

Participant N, Community Scientist

## 4. Discussion

Participants identified barriers in knowledge, relative priority, and ability to access lung cancer screening. Participants affirmed that a clinical community navigator model could be used to incorporate their suggested patient-facing implementation strategies by redesigning the LDCT-LCS workflow and helping patients with many of the logistical barriers to screening. Participants suggested additional provider-facing implementation strategies including educational meetings, clinical champions, and providing feedback on clinic specific LDCT-LCS screening rates.

Patient-facing logistical and knowledge barriers overlapped with those commonly identified in the literature. In a 2022 review using CFIR, patient needs and resources (i.e., costs, transportation, and time) along with patients’ knowledge were commonly reported patient-level barriers [41]. In this review, costs typically referred to either the cost to obtain insurance or the cost of co-pays in studies that occurred prior to USPSTF expanding the eligibility criteria. Since private insurers are required to cover Grade A and B USPSTF recommendations, which include LDCT-LCS, with no cost-sharing [42], it is notable that participants still identified this as a major concern in our study, especially in a population already engaged in primary care, with patients who presumably have insurance. Participants also described fatalistic beliefs about lung cancer prognosis among patients, which aligns with a growing body of evidence linking lung cancer stigma and nihilism to reduced screening uptake and delayed care-seeking [43,44,45,46,47]. Addressing these beliefs may require messaging strategies that go beyond informational content to actively counter stigma-related misconceptions about treatability and survivorship, similar to those used in LungTalk. On the provider side, the discrepancy between private insurance coverage and Medicare insurance coverage may be contributing to this confusion, as a number of providers mentioned the difference, but none were able to correctly remember which populations were covered [48]. This is in line with previous surveys which have shown that while providers are aware of USPSTF guidelines, it is difficult for many to recall the details of eligibility and coverage [49].

Participants felt that gaps in patients’ knowledge about their eligibility and the lack of cost-sharing for screening could be addressed through the clinical community navigator model. Participants suggested that clinical community navigators could promote access by helping patients navigate the understaffed scheduling system for LDCT. Additionally, participants thought that patients would be more receptive to the clinical community navigators’ contact if they linked it to patients’ existing relationships with their primary care providers, making it clear that the provider supported lung cancer screening. This aligns with a previous review, which found that a provider recommendation to screen was a major facilitator to patients’ willingness to complete LDCT-LCS [9].

Participants were especially concerned with transportation and distance to LDCT for rural patients, who would often have to travel over an hour each way to access LDCT scans. Increased distance to screening is a common barrier in southeastern states, which have both the highest burden of lung cancer and fewest number of facilities for LDCT-LCS per 100,000 people [49,50]. In the health system’s catchment area, insurers, including Florida Medicaid providers, will reimburse or provide transportation through ride-share services, and local organizations provide shuttles. However, participants suggested that in reality, the scheduling and reimbursement for these services were more limited, especially in rural areas, hampering their impact. Interestingly, some participants suggested that it was not purely the distance, but also the actual facilities, which are located on a large hospital campus in a high-traffic part of Gainesville and which is known locally for its confusing parking system. One participant suggested that making it clear that there were also two suburban LDCT locations, which while only marginally closer to rural patients in terms of distance, were much easier for them to navigate, could improve uptake for patients. Participants also suggested that clinical community navigators could connect patients to transportation vouchers, which could reimburse patients for gas or use of a ride-share app, helping improve access.

While the clinical community navigator strategy was perceived to be an effective way to address patient knowledge, scheduling difficulties, confusion over cost, and provider time, it does not address some of the barriers due to IT infrastructure. Poor smoking documentation in the EMR is a pervasive issue across health systems [13,51]. Researchers at UF have developed a natural language processing model that uses unstructured data from clinical notes to more accurately identify eligible patients within our EMR [52]. Adding this feature to patient identification within the clinical community navigator pilot could help address this barrier. Patients may still encounter barriers related to technological literacy, though participants in our study were divided over whether this would be a barrier for most patients. Some participants were concerned that technological literacy might limit older adults’ ability to participate, while others, including community scientists who were themselves older, believed that EMR use was common among older adults. Research by the University of Michigan National Poll on Healthy Aging suggests that EMR usage has increased considerably among older adults following the COVID-19 pandemic and resulting shift to telehealth [53]. Their polling showed that 51% of adults aged 50–80 had an EMR account set up in March 2018, but that proportion had increased to 78% by January 2023, with 85% of EMR users having accessed their account in the last 6 months and 57% feeling “very confident” about navigating their EMR. This increase in use may indicate that the EMR-based interventions are a feasible way to reach adults eligible for LDCT-LCS.

Participants in this study suggested that relative priority was a major barrier for both providers and patients. This is consistent with a 2022 study that found providers were significantly less likely to endorse LDCT-LCS as very effective compared with colonoscopy, mammography, or cervical cancer screening [54]. Lack of confidence in clinical evidence, confusion about guidelines, and confusion about referral pathways are all associated with decreased likelihood of referring patients for LDCT [55]. Providers found the clinical community navigator strategy appropriate and thought it could circumvent the limited time and other priorities within a typical primary care visit. In addition to this new workflow, participants also suggested selecting clinical champions, providing information on screening rates, and educational meetings. Providers in this study often perceived the screening rates at their clinics to be much higher than institutional data suggested, so providing clinic- or provider-level data on screening could certainly help address these assumptions. Educational meetings were thought to have the dual benefit of supporting implementation of the intervention and providing a chance to reiterate eligibility criteria and insurance coverage.

Our study is not without limitations. While participants were recruited purposively until no new sub-themes emerged, it is possible that other providers, staff, or community scientists within the UF Health system may have offered differing perspectives from participants. Furthermore, while selecting all participants from this health system was intentional to inform the development of a clinical community navigator pilot, the barriers and strategies identified here may differ meaningfully from those in other health systems. Importantly, our sample did not include individuals eligible for screening who are not currently engaged in primary care, limiting the generalizability of our findings. Future studies could evaluate barriers and strategies to aid implementation of a clinical community navigator strategy in other settings.

## 5. Conclusions

Participants recognized knowledge, IT infrastructure, and relative priority as barriers to LDCT-LCS on both the patient and provider level. Limited staffing levels in radiology also contributed to significant reported difficulty scheduling LDCT scans. Rural patients may face additional barriers to uptake of lung cancer screening, including internet access and distance to LDCT. A clinical community navigator model can address many of the logistical challenges of patient screening and simplify the workflow for providers. Ensuring that navigators are clear about insurance coverage and build on existing patient–provider relationships can help make the strategy more effective for patients. Building in clinical champions, providing education on eligibility and coverage, and clarifying current LDCT-LCS screening rates can help get providers’ buy-in.

## Figures and Tables

**Table 1:** CFIR barriers to lung cancer screening and corresponding ERIC strategies to support implementation.

CFIR Domain	CFIR Construct	Example		Corresponding ERIC Strategy	Example
Outer Setting	Patient needs and resources *^+^	Time off work, cost, travel	→	Address patient access *^+^	Travel voucher, weekend clinics, clarify insurance coverage
	Financing *^+^	Confusion over insurance coverage			
Inner Setting	Physical infrastructure *^+^	Distance to CT scanner			
	IT infrastructure *^+^	Smoking documentation			
	Work infrastructure *^+^	Staffing, scheduling process	→	Assess and redesign workflows	Clinical community navigators
	Relative priority *^+^	Busy primary care visit	→	Audit and provide feedback *	Information on current screening rates
	Access to knowledge and information *^+^	Provider awareness of eligibility, patient awareness of eligibility	→	Identify and prepare clinical champions *	
				Conduct educational meetings *^+^	Educate on eligibility
				Raise patient awareness of intervention *^+^	Health education materials

* Primary care providers, clinical community navigators, or radiology staff noted. ^+^ Community scientists noted. Examples are selected from barriers and strategies suggested by participants.

## Data Availability

Due to privacy concerns, data is not publicly available. De-identified interviews will be made available upon reasonable request.

## Supplementary Materials

The following supporting information can be downloaded at: https://www.mdpi.com/article/10.3390/curroncol33090499/s1, File S1: Interview Guide Providers and Staff; File S2: Interview Guide Community Scientists.

## Abbreviations

The following abbreviations are used in this manuscript:

EMR	Electronic medical record
LDCT-LCS	Low-dose computed tomography for lung cancer screening
IT	Information technology
UF	University of Florida
USPSTF	US Preventative Services Task Force
