# Supplementary material for: Identifying Barriers and Strategies to Support a Community Navigator-Driven Approach for Lung Cancer Screening
Source: Curr Oncol. 2026 Aug 24;33(9):499. doi: 10.3390/curroncol33090499 (PMC13605281; doi:10.3390/curroncol33090499)
Supplement: Supplementary file 1 [file curroncol-33-00499-s001.zip › File S1-LCS Interview Guide V3 - Providers.pdf]

LCS SDM Interview Guide
Participant Study ID:
Interviewer:
Date of interview:

Thank you for participating. In this interview, we aim to learn about barriers and facilitators to lung cancer screening. If we have your permission, we would like to record this interview for note taking purposes. The information you share with us today will be used to summarize experiences and may be used to inform recommendations. No identifying information will be shared in any publications or other reporting that we do.

I. Perceived Barriers

To start, I have a few broad questions about the barriers you see to lung cancer screening uptake, including barriers at the organizational/system, provider, and patient level.

1. How do you approach lung cancer screening with your patients?

2. Overall, what do you perceive to be the biggest barrier to lung cancer screening for eligible individuals throughout UF Health?
   *If needed, can prompt about barriers at the organizational/system, provider, and patient level.

3. What do you think is the biggest barrier to <u>clinicians</u> recommending lung cancer screening? What about the biggest facilitator?
   - *Prompt: knowledge of eligibility, quality of evidence, time pressure, other competing priorities, concerns about follow-up*
   - **What challenges do clinicians experience when communicating about LCS with patients, if any?*

4. What is the biggest barrier you perceive to <u>patients</u> completing lung cancer screening? What about the biggest facilitator?
   - *Prompt: perceived risk, knowledge of LCS, logistics (cost, travel, time off work), lack of provider endorsement*
   - **What challenges do patients experience when communicating about LCS with clinicians, if any?*

5. What do you think is the biggest <u>organizational</u> barrier to lung cancer screening throughout UF Health? What about the biggest facilitator?
   - *Prompt: reimbursement policy, staffing levels, EHR documentation and reminders, clarity about referral process*

6. What about any larger <u>systems-level</u> barriers to lung cancer screening? What about the biggest facilitator?
   - *Prompt: state or national policies, insurance/CMS issues, etc.*

7. Overall, which of the barriers that we've discussed do you see as the largest barrier for eligible patients to get screened for lung cancer? Which barrier is most important to address to increase lung cancer screening rates among eligible individuals? Which barrier is the most feasible to address?
    - *Prompt: Do you think this barrier should be prioritized first in addressing LCS? Why/why not?*

II. Strategy to be tested (LungTalk)

We are piloting a new pathway to help improve patient's knowledge about lung cancer screening and reduce logistic barriers to their screening.

Potentially eligible patients (identified through the electronic health record, if they have any smoking history and are between the ages 50-80 years old) will be sent a shared-decision making tool via MyChart and will be asked if they are interested in lung cancer screening.

If they are, they will be prompted to confirm their smoking history. If they are eligible for screening (USPSTF guidelines: 50-80 years old, 20 years pack history, quit date <15 years), a CCN will pend an order to their primary care provider and help patients address barriers to screening (cost, transportation).

8. What feedback do you have about this strategy to increase LCS among eligible persons?
    - *Prompt: Do you have any concerns or suggestions about this potential strategy?*
    - *Prompt: patient use of MyChart, documentation of smoking in the health record, logistics of referral/follow up*
9. In order to test the strategy described above, we may need to implement a few changes to primary care / family medicine clinical workflows. Specifically, we will need to work with providers to place orders for LCS for patients who receive LungTalk and want to then do lung cancer screening.
   How do you think this could best be integrated into practice?

III. Potential Implementation Strategies

Thanks for your feedback on how to integrate LungTalk.

10. You mentioned barriers related to XYZ. Can you think of any strategies to address these?

11. Do you have any other strategies that you can think of or would like to suggest to improve LCS at UF Health?

    What about …

    - Identifying clinical champions to advocate for the program
    - Educational trainings or materials
        - For referring providers, community health workers, nurse navigators, or rooming staff (improve smoking documentation)?
        - What would be the best way to deliver it?

- Audit and provide iterative feedback
    - For referring providers, community health workers, nurse navigators, or rooming staff?
- Peer-peer knowledge sharing
    - For referring providers, community health workers, nurse navigators, or rooming staff?
- Broader lung cancer screening promotion – email promotion from UF Health Cancer Center and departmental newsletters
- Regular check-ins/reminders with clinics
    - Over email, in-person

12. Which of the implementation strategies that I mentioned do you think would be most helpful, if any? Why or why not?

Thank you again! I really appreciate the time you took to talk to me today.
