# Supplementary material for: Identifying Barriers and Strategies to Support a Community Navigator-Driven Approach for Lung Cancer Screening
Source: Curr Oncol. 2026 Aug 24;33(9):499. doi: 10.3390/curroncol33090499 (PMC13605281; doi:10.3390/curroncol33090499)
Supplement: Supplementary file 1 [file curroncol-33-00499-s001.zip › File S2-LCS Interview Guide V2 - Patients.pdf]

LCS SDM Interview Guide
Participant Study ID:
Interviewer:
Date of interview:

Thank you for participating. In this interview, we aim to learn about barriers and facilitators to lung cancer screening. If we have your permission, we would like to record this interview for note taking purposes. The information you share with us today will be used to summarize experiences and may be used to inform recommendations. No identifying information will be shared in any publications or other reporting that we do.

I. Perceived Barriers

To start, I have a few broad questions about the barriers to lung cancer screening uptake,

1. What have your own experiences been with cancer screening?

2. Overall, what do you think are the biggest barriers to lung cancer screening UF Health? What about facilitators?

    - What do you think are the biggest barriers patients have in completing lung cancer screening? What about facilitators?
    - *Prompt: perceived risk, knowledge of LCS, logistics (cost, travel, time off work), lack of provider endorsement*
    - **What challenges do patients experience when communicating about LCS with clinicians, if any?*

    - [if necessary] What do you think is the biggest organizational barrier to lung cancer screening throughout UF Health? What about the biggest facilitator?
        - *Prompt: reimbursement policy, staffing levels, EHR documentation and reminders, clarity about referral process*

    - [if necessary] What about any larger systems-level barriers to lung cancer screening? What about the biggest facilitator?
        - *Prompt: state or national policies, insurance/CMS issues, etc.*

3. Overall, which of the barriers that we've discussed do you see as the largest barrier to lung cancer screening? Which barrier is most important to address to increase lung cancer screening rates among eligible individuals? And why? Which barrier is the most feasible to address? And why?
    - *Prompt: Do you think this barrier should be prioritized first in addressing LCS? Why/why not?*

II. Strategy to be tested (LungTalk)

We are piloting a new pathway to help improve patient's knowledge about lung cancer screening and reduce logistic barriers to their screening.

Potentially eligible patients (identified through the electronic health record, if they have any smoking history and are between the ages 50-80 years old) will be sent a shared-decision making tool via MyChart and will be asked if they are interested in lung cancer screening.

If they are, they will be prompted to confirm their smoking history. If they are eligible for screening (USPSTF guidelines: 50-80 years old, 20 years pack history, quit date <15 years), a CCN will pend an order to their primary care provider and help patients address barriers to screening (cost, transportation).

4. What do you think about this strategy to increase LCS among eligible persons?
    - *Prompt: Do you have any concerns or suggestions about this potential strategy?*
    - *Prompt: patient use of MyChart, documentation of smoking in the health record, logistics of referral/follow up*

III. Potential Implementation Strategies

Thanks for your feedback on how to implement this strategy.

5. You mentioned barriers related to XYZ. What strategies might address these?

6. What other strategies might improve LCS at UF Health?

    What about …

    - Offering weekend appointments for LDCT scheduling
    - Check-in to ensure that appointment was scheduled
    - Broader promotion of lung cancer screening (flyers, emails, social media)
    - Community health navigator help with :
        - Insurance
        - Transportation

7. Which of the implementation strategies that I mentioned do you think would be most helpful, if any? Why or why not?

Thank you again! I really appreciate the time you took to talk to me today.
